# Synthesis, Characterization and Antiproliferative Evaluation of Pt(II) and Pd(II) Complexes with a Thiazine-Pyridine Derivative Ligand †

**DOI:** 10.3390/ph14050395

**Published:** 2021-04-22

**Authors:** Silvia Gutiérrez-Tarriño, Javier Espino, Francisco Luna-Giles, Ana B. Rodríguez, José A. Pariente, Emilio Viñuelas-Zahínos

**Affiliations:** 1 Coordination Chemistry Research Group, Department of Organic and Inorganic Chemistry, Faculty of Science, University of Extremadura, 06006 Badajoz, Spain; silgutar@doctor.upv.es (S.G.-T.); pacoluna@unex.es (F.L.-G.); 2 Neuroimmunophysiology and Chrononutrition Research Group, Department of Physiology, Faculty of Science, University of Extremadura, 06006 Badajoz, Spain; moratino@unex.es (A.B.R.); pariente@unex.es (J.A.P.)

**Keywords:** Pd(II) and Pt(II) complexes, thiazine, pyridine, cytotoxicity, tumor cells, U-937, HL-60, SK-OV-3, HeLa

## Abstract

Chemical, pharmacological, and clinical research on anticancer coordination complexes has led to noteworthy anticancer drugs such as cisplatin, carboplatin and oxaliplatin. Although these compounds are effective chemotherapeutic agents in the treatment of different tumors, they are associated with high toxicity and numerous side effects. Several studies have shown that the range of platinum complexes with antitumor activity is not limited to structural analogs of cisplatin. Therefore, the development of convenient anticancer drugs that can be effectively used for the treatment of human tumors has become the main goal of most research groups in this field. In this sense, active platinum complexes without NH groups, transplatinum complexes, multinuclear complexes, cationic complexes, and several classes of palladium(II) complexes have emerged. Herein, the synthesis and characterization of two Pt(II) or Pd(II) complexes with PyTz (2-(2-pyridyl)iminotetrahydro-1,3-thiazine), a thiazine derivative ligand, with the formula [MCl_2_(PyTz)]·C_2_H_6_O (M = Pt(II) or Pd(II)) were reported. The potential anticancer ability of both complexes was evaluated in epithelial cervix carcinoma HeLa, human ovary adenocarcinoma SK-OV-3, human histiocytic lymphoma U-937, and human promyelocytic leukemia HL-60 cell lines. Interestingly, the Pt(II) complex showed great cytotoxic potential against all tumor cell lines tested, whereas the Pd(II) complex displayed slight antitumor actions.

## 1. Introduction

In the last few years, many promising advances have been achieved in the field of cancer treatment. However, the disease still widely affects humanity and remains to be the second leading cause of death after cardiovascular diseases [1]. A total of 8.8 million people died from cancer in 2015, and the number of new cases is expected to increase by approximately 70% over the next twenty years [2]. Therefore, it is necessary to keep investigating the design of novel antitumor agents and seek new approaches.

Cisplatin (*cis*-diamminedichloroplatinum(II)), approved in 1978 for clinical use, is one of the most widely used complexes in anticancer therapy together with second-generation Pt complexes such as carboplatin (diammine(1,1-cyclobutanedicarboxylato(2-)-O,O′)platinum(II)), approved in 1993, or oxaliplatin (cis-[(*1R,2R*)-1,2-cyclohexanediammine-N,N′][oxalato(2-)-O,O′]platinum(II)), approved in 2002 [3]. The square-planar Pt(II) complexes are activated by slow hydrolysis of the anionic ligands and the corresponding cationic aquo complexes formed bind to DNA creating stable intrachain cross-links that block replication and/or prevent transcription [4].

Although cisplatin is used against several kinds of cancer, such as cervical, bladder, lung, neck and head cancers [5,6,7], the use and efficacy of this drug are limited because of the intrinsic and acquired cell resistance after extended treatment [8,9], as well as due to a large number of side effects [10,11,12] such as nausea, vomiting, ototoxicity, neurotoxicity, hemolytic anemia, nephrotoxicity and electrolyte disturbance. Even though carboplatin and oxaliplatin were introduced to overcome the disadvantages of cisplatin, this has not been completely achieved [13], thus indicating that it is still necessary to develop new compounds to be used at lower therapeutic doses with milder toxicity.

In the last two decades, several research groups have focused on obtaining metal-based drugs with high anticancer activity and reduced side effects with respect to cisplatin and its analogues. The significant similarity between the coordination chemistry of Pd(II) and Pt(II) complexes has promoted studies on Pd(II) compounds as anticancer drugs [14,15]. The ligand exchange rate of Pd(II) complexes is about 10^4^–10^5^ times faster than the one for Pt(II) analogues, but they present better solubility compared to Pt(II) complexes [15]. Previous works have reported that a correct selection of ligands is very important, since they play a key role in several issues such as reactivity, lipophilicity and stability [16]. In this sense, different Pd(II) complexes with promising antitumor activity have been reported [14,17,18,19].

Nowadays, new coordination complexes are obtained by adding bioactive ligands such as heterocycles containing N- and S-donor atoms in their structure. Among these heterocycles, thiazines are six-membered heterocycles containing a N atom and a S atom. Depending on the relative position of the two heteroatoms and the degree of oxidation of the ring system, a large number of isomeric structures are possible. Some thiazines are well known, but others are rare and appear only as intermediates. There are some compounds derived from thiazines, such as N-(5,6-dihydro-4H-1,3-thiazin-2-yl)benzamide bromohydrate, which shows a high cytotoxic activity on human leukemic cells [20]. Moreover, this compound has the advantage that it is not toxic to healthy lymphocytes. This cytotoxic activity is related to the inhibitory capacity of this compound on nitric oxide synthase (NOS), an enzyme that regulates endogenous NO synthesis, which has a modulatory character in apoptosis or cell death [20].

On the other hand, pyridine is one of the best-known heterocyclic nitrogen ligands, and its coordination chemistry has been studied in depth. In fact, the Cambridge Structural Database (CSD, Conquest Version V5.41, Aug2020) [21] lists around 150,000 coordination compounds between a metal and a pyridine ring, of which 5313 complexes bear a Pt(II)-pyridine bond and 5757 present a Pd(II)-pyridine bond. It should be noted that a large number of these compounds show recognized antitumor activity both in vitro and in vivo. For example, picoplatin (*cis*-aminedichloro(2-methilpyridine)platinum(II)) has been evaluated in lung, colon, prostate and ovarian cancer cells, showing high efficiency and low toxicity [22], while *trans*-dichloro[2-(hydroxyalkyl)pyridine] palladium(II) complexes show activity against lung and colon cancer cells, as well as in sarcomas [14],

In this sense, the ligand 2-(2-pyridyl)iminotetrahydro-1,3-thiazine (PyTz), which contains a 1,3-thiazine ring and a pyridine ring in its structure and has been previously used in our group for the synthesis of several transition metal complexes [23,24,25], could be a promising ligand in the synthesis of Pt(II) and Pd(II) complexes with antitumor activity.

Therefore, in this work, the synthesis, structural characterization and evaluation of cytotoxic potential against four human tumor cells of Pt(II) and Pd(II) complexes with PyTz was carried out. Moreover, the crystal structure of 2-(2-pyridyl)iminotetrahydro-1,3-thiazinehydrochloride–water (1/2) (PyTzHCl·2H_2_O), which has never been described in the literature, is presented herein. Likewise, it is reported that PyTz-containing Pt(II) complex is considerably more biologically active than its Pd(II) counterpart.

## 2. Results and Discussion

### 2.1. Crystal Structures

**Figure 1:**
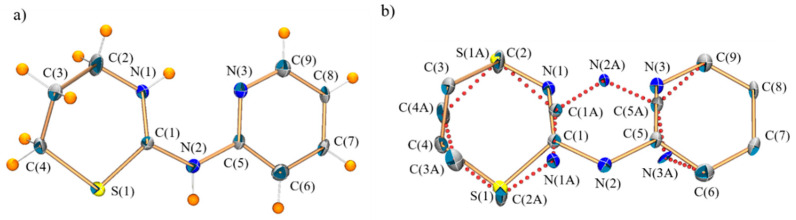
(**a**) Crystal structure and numbering system of majority arrangement for PyTzHCl·2H_2_O, (**b**) Resolution of static disorder in the crystal structure and numbering system for PyTzHCl·2H_2_O.

The X-ray study revealed that the PyTz·HCl crystals are made up of triclinic unit cells, each containing two chloride ions, two PyTzH^+^ cations and four water molecules of crystallization. It could be therefore formulated as PyTz·HCl·2H_2_O. A diagram of the molecular structure and the atom numbering system used is shown in Figure 1a. Selected bond lengths, angles and hydrogen-bond parameters are listed in Table 1.

In the electronic density map obtained in the resolution of the crystalline structure, it was possible to verify that the atoms of the organic cation were involved in a static disorder and, consequently, they were modeled using two sets of positions, for which a normal numbering system was used for the atoms of one of the arrangements and the letter A was added to those of the other (Figure 1b). However, it was not possible to model all the atoms of arrangement A because some of them almost matched in space with some atoms of the pyridine ring and, in addition, this led to an invalid model in which the pyridine was not flat. In this sense, the occupancy of the two alternative orientations was restricted to the unit by calculating the occupancy factors, which were found to be 0.82(1) for the numbered orientation and 0.18(1) for the orientation named as A.

For this compound, as can be observed in Table 1, the endocyclic bond C(1)-N(1) length [1.302(2) Å] is similar to the one of the exocyclic bond C(1)-N(2) [1.355(2) Å], both being between C=N [1.26 Å] and C-N [1.47 Å] [7]. It can also be observed that the length C(1)-S(1) [1.744(2) Å] is smaller than the length C(4)-S(1) [1.806(2) Å] and it is between C=S (1.61 Å) and C-S (1.82 Å) [26]. On the other hand, bond angles in which the N(1), C(1), N(2) and S(1) atoms are involved showed that the hybridization adopted for the first three is ~sp^2^, while sulfur uses ~p orbitals to build the σ structure. Moreover, all these atoms are coplanar. This information indicates the presence of an extended multiple bond around the C(1) atom. These remarks, together with the presence of a hydrogen atom linked to N(1), support the fact that the predominant tautomeric form is the iminotetrahydro-1,3-thiazine form instead of the amino-5,6-dihydro-4H-1,3-thiazine form, for which a shorter exocyclic C-N bond length and a significantly longer endocyclic C-N distance would be expected.

**Figure 2:**
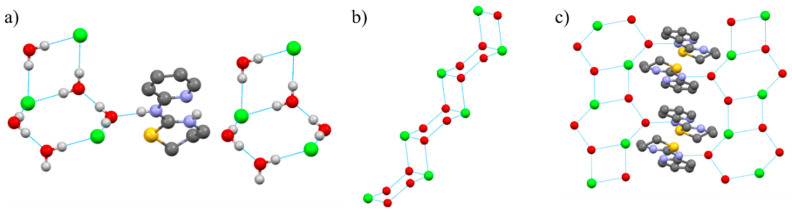
(**a**) Intermolecular hydrogen bond system in the crystal structure of PyTzHCl·2H_2_O. (**b**) Conformation of {[(H_2_O)_5_Cl_3_]^3−^}_n_ tapes in the PyTzHCl·2H_2_O crystal structure. (**c**) Supramolecular structure of PyTzHCl·2H_2_O.

Interestingly, as can be seen in Figure 2a, the crystal is stabilized by an intermolecular hydrogen bond system in which each chloride ion behaves like a hydrogen acceptor, N(2) atom as a hydrogen donator and water behaves in both ways.

In particular, the chloride ions and the water molecules create {[(H_2_O)_5_Cl_3_]^3−^}_n_ tapes, whose structural parameters are summarized in Figure S1. In these tapes, the planar [(H_2_O)_2_Cl_2_]^2−^ units are linked by two water molecules, creating staircase-like architectures running parallel to the *a* axis (Figure 2b). Alternatively, they can be described as a periodic repetition of fused four-membered rings (2 H_2_O and 2 Cl^−^) and six-membered rings (4 H_2_O and 2 Cl^−^) in the tape, which can be considered to come from the T4(2)6(2) water assembly with two H_2_O molecules from the hexagonal ring substituted by two chloride ions. The four-membered ring is planar but, in the (H_2_O)_4_Cl_2_ ring, two of the water molecules are found at 1.003 Å away from the best least-squared plane, adopting a chair configuration (Figure 2b). Furthermore, the water molecules that link the planar [(H_2_O)_2_Cl_2_]^2−^ units are hydrogen bonded to an NH fragment of an organic cation PyTzH^+^, these cations filling in the spaces formed between two chains (Figure 2c).

**Figure 3:**
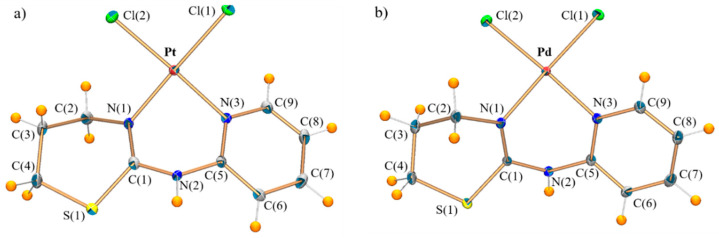
Crystal structures of (**a**) PtPyTz and (**b**) PdPyTz. Ethanol solvate is omitted for clarity.

The crystal structures of metal complexes are made up of monomeric [MCl_2_(PyTz)] molecules (Figure 3) and ethanol solvate molecules, therefore the compounds can be formulated as [PtCl_2_(PyTz)]·C_2_H_6_O (PtPyTz) and [PdCl_2_(PyTz)]·C_2_H_6_O (PdPyTz). As can be seen, PtPyTz and PdPyTz have the same coordination mode, with the metal center located in a slightly distorted square-planar coordination geometry (dihedral angles between planes Cl(1)-M-Cl(2) and N(1)-M-N(3) are 2.6° in PtPyTz and 3.6° in PdPyTz). This metal center is surrounded by one PyTz molecule, which behaves as a chelated bidentate ligand coordinated by one pyridinic nitrogen and one thiazinic nitrogen, thus forming a six-membered metallocycle with a near to boat configuration in both complexes. Two chlorine ions in *cis* disposition complete the coordination environment.

As can be seen in Table 2, M-Cl bond lengths in both complexes are similar to calculated mean values for squared-planar cis complexes with a Cl_2_N_2_ coordination environment around M(II) ion (2.298(18) Å for 620 Pt(II) complexes; 2.293(19) Å for 879 Pd(II) complexes) obtained with CONQUEST software from the Cambridge Structural Database (CSD, Version V5.41, Aug2020) [21]. In the same way, the M-N_pyridine_ bond lengths are similar to the average value found for squared-planar complexes with Cl_2_N_2_ coordination sphere around M(II) ion (2.020(23) Å for 315 Pt(II) complexes; 2.039(28) Å for 427 Pd(II) complexes) in CSD [21]. However, the Pt-N_thiazine_ bond distance is slightly shorter than the mean value calculated [2.091(32) Å] for five crystal structures with this type of bond in CSD [21]. Finally, to our best knowledge, PdPyTz is the first crystal structure of a Pd(II) complex having a 1,3-thiazine link to Pd(II) in CSD [21].

With respect to the supramolecular arrangement, the structure is stabilized by intramolecular hydrogen bonds between the oxygen atom from the ethanol and the hydrogen atom bonded to N(2), and between the O-H of the ethanol and Cl(2), building chains along axis *b* (Figures S2 and S3 for PtPyTz and PdPyTz, respectively).

### 2.2. Spectroscopic Studies

The ligand PyTz and its Pt(II) and Pd(II) complexes were studied by spectroscopic techniques such as NMR and IR. ^1^H and ^13^C NMR spectra of the ligand and both complexes are represented in Figures S4–S9 and the ^1^H-NMR spectral data for PyTz and its complexes are shown in Table S1. As can be observed, the signals in metal complexes are shifted downfield compared to those corresponding to the free ligand. These differences indicate that the organic ligand is coordinated to the metal center in the *N,N*-dimethylformamide (DMF) solution for both complexes. Moreover, both the ligand and the complexes were kept in solution for 25 days before being measured by NMR again, which shows the high stability of these complexes in the DMF medium. Finally, the stability of compounds in aqueous media was determined by ^1^H-NMR spectroscopy adding 0.55 mL of D_2_O at a 50 µL of solution of the compound in DMF-d_7_. Spectra of compounds (Figures S10–S12) are identical after 24 h for PyTz and PdPyTz, which indicates that compounds stay unalterable. However, for PtPyTz the apparition of new signals, clearly after 6 h, shows a certain degree of decomposition.

The IR spectra of complexes PtPyTz and PdPyTz (Figures S13–S16) showed several strong-medium ν(NH) absorption between 3262 and 3126 cm^−1^, while only a medium band at 3181 cm^−1^ was observed for the uncoordinated PyTz ligand. Moreover, a strong ν(C=N) absorption (at 1631 cm^−1^ in PtPyTz and at 1629 cm^−1^ in PdPyTz) could be observed. These bands were shifted to higher wavenumbers relative to the imine function of the free ligand (1593 cm^−1^). Both facts could be correlated with the change of tautomeric form from iminotetrahydro-1,3-thiazine for the free ligand to the amino-5,6-dihydro-4H-1,3-thiazine when the ligand is coordinated to a metallic center. Likewise, the bands corresponding to the in-plane stretching vibrations of the pyridine ring were also shifted, in general, to lower wavenumbers compared with the ones of the uncoordinated ligand. Consequently, coordination via the nitrogen atom of the pyridine ring of the ligand can be deduced.

In the low-frequency region, the C1 symmetry of Pt(II) and Pd(II) complexes predicted the appearance of four bands assignable to metal–ligand stretching vibrations, ν(M-ligand) in IR [27,28]. However, for cis complexes, it could be expected two vibrations ν(M-Cl) corresponding to symmetric and asymmetric stretching mode (314 and 328 cm^−1^ for PtPyTz, 323 and 330 cm^−1^ for PdPyTz) [29,30,31,32,33]. Likewise, in this type of complexes, two bands for each vibration ν(M-N) are expected, although it can be normally only observed one of them. In this case, the bands which appeared at 259 and 252 cm^−1^ in PtPyTz and at 254 cm^−1^ in PdPyTz spectra were attributed to ν(M-N_pyridine_) vibrations [29], while the bands detected at 243 and 232 cm^−1^ in PtPyTz and at 242 and 233 cm^−1^ in PdPyTz were assigned to ν(M-N_thiazine_) vibrations [23,24].

### 2.3. In Silico ADME Prediction

The SwissADME web tool was used to compute physiochemical properties and the Absorption, Distribution, Metabolism, and Excretion (ADME) parameters [34]. From this computation, the pharmacokinetics and drug-likeness of compounds were predicted. The obtained results are presented in Table S2. It should be noted that the compounds were evaluated according to Lipinski’s rule of five [35] and PAINS alerts [36], having zero violations or alerts. When compared to the allowed values for polarity (TPSA ≤ 140 Å^2^), lipophilicity (logP_o/w_ < 5), solubility (log s ≥ −6), size (M_w_ ≤ 500 g/mol) and flexibility (number of rotable bonds ≤ 9), all compounds show calculated values within these ranges. Finally, compounds showed a high gastrointestinal absorption potential and blood–brain barrier crossing ability. These results lead to the conclusion that the investigated compounds could be good candidates as anticancer drugs.

### 2.4. Biological Activity

**Figure 4:**
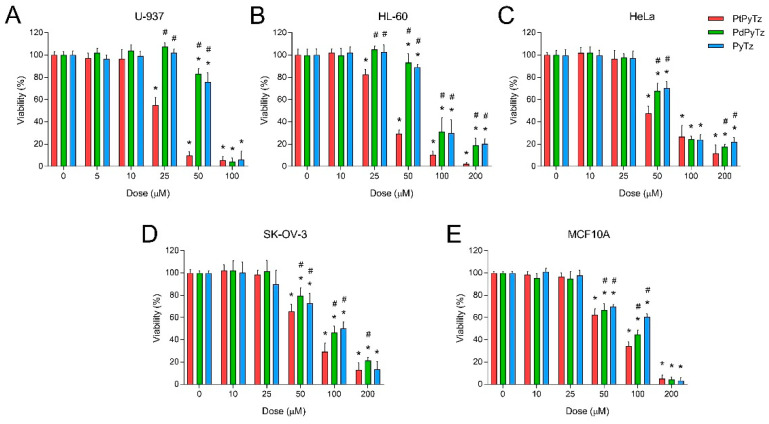
Dose–response curves of chemotherapeutics on cell viability of tumor and non-tumor cell lines. (**A**) U-937, (**B**) HL-60, (**C**) HeLa, (**D**) SK-OV-3 and (**E**) MCF10A cells were treated for 24 h with increasing concentrations (5, 10, 25, 50, 100 and 200 μM, as indicated) of the ligand PyTz, the Pt(II) (PtPyTz) and Pd(II) (PdPyTz) complexes, or the vehicle (DMF, control). Data represent means ± S.D. of six independent experiments and are expressed as a percentage of control values. * *p* < 0.05 compared to their corresponding control values. ^#^ *p* < 0.05 compared to their corresponding PtPyTz values.

Finally, the cytotoxic potential of the ligand PyTz and its Pt(II) and Pd(II) complexes was evaluated against four selected human tumor cell lines, namely U-937 histiocytic lymphoma, HL-60 promyelocytic leukemia, HeLa epithelial cervix carcinoma, and SK-OV-3 ovary adenocarcinoma cells (Figure 4).

The Pt(II) complex PtPyTz proved to be more promising as an anticancer drug since it depicted great cytotoxic potential against all tumor cell lines tested (Figure 4). The Pt(II) complex was more selective towards leukemic cell lines U-937 and HL-60 (IC_50_ values were 26.36 ± 2.56 µM and 39.25 ± 3.86 µM, respectively; Table 3), and a bit less efficient against solid tumor cell lines HeLa and SK-OV-3 (IC_50_ values were 63.38 ± 5.63 µM and 77.97 ± 6.36 µM, respectively; Table 3). On the contrary, the Pd(II) complex PdPyTz showed low cytotoxic potential, the most remarkable cell killing ability being displayed against HeLa cells (IC_50_ values were 78.62 ± 8.21 µM; Table 3 and Figure 4). The effect of the ligand PyTz was comparable to that of PdPyTz in all four tumor cell lines (Figure 4 and Table 3), which confirms the slight antitumor actions of the Pd(II) complex. Likewise, both PtPyTz and PdPyTz showed moderate toxicity against epithelial non-tumor MCF10A cell line (IC_50_ values were 75.94 ± 7.74 µM and 81.54 ± 8.29 µM, respectively; Table 3 and Figure 4); however, the effective doses of Pt(II) complex, especially those against leukemic cell lines U-937 and HL-60 (26.36 and 39.25 µM, respectively), had negligible effects on cell viability of MCF10A epithelial cells. In any case, the standard chemotherapeutic agent cisplatin exhibited lower IC_50_ values than the PyTz-containing complexes towards the four tumor cell lines (Table 3), although it also showed higher toxicity against MCF10A non-tumor cells (Table 3). The fact that Pt(II) complexes are considerably more active than their Pd(II) counterparts has been previously reported for different organometallic compounds, such as pyrazole-based complexes [37] and complexes with tertiary arsenic ligands [38]. Nonetheless, other studies have reported Pd(II) complexes with both superior [39] and similar [2,40] biological activity to that of analogue Pt(II) complexes, thereby suggesting that the cytotoxic effect can be modulated by either the central metal or the ligand (the former being the case in the present study).

## 3. Materials and Methods

### 3.1. General Procedures

Human epithelial cervix carcinoma HeLa cell line (No. 93021013), human ovary adenocarcinoma SK-OV-3 cell line (No. 91091004), human histiocytic lymphoma U-937 cell line (No. 85011440), and human promyelocytic leukemia HL-60 15-12 cell line (No. 88120805) were purchased from the European Collection of Authenticated Cell Cultures (ECACC) (Dorset, U.K.). Human non-tumorigenic epithelial MCF10A cell line (No. CRL-10317) was obtained from the American Type Culture Collection (ATCC) (Barcelona, Spain). RPMI-1640, McCoy′s 5a, Dulbecco’s modified Eagle’s medium (DMEM), DMEM F-12, penicillin/streptomycin, foetal bovine serum (FBS), horse serum and L-glutamine were procured from ThermoFisher Scientific (Barcelona, Spain). Hydrocortisone, insulin, epidermal growth factor (EGF) and cholera toxin were bought from Sigma (Madrid, Spain). CellTiter 96 AQueous One Solution Cell Proliferation Assay was acquired from Promega (Madrid, Spain). All reagents and solvents were purchased from commercial suppliers and used without further purification.

On the other hand, 2-(2-pyridyl)iminotetrahydro-1,3-thiazinehydrochloride–water (1/2) (PyTzHCl·2H_2_O), the ligand 2-(2-pyridyl)iminotetrahydro-1,3-thiazine (PyTz) [24] and the precursor [PtCl_2_(DMSO)_2_] [41] were prepared as described in the literature.

Isolated compounds were characterized by elemental analysis, ^1^H-NMR, ^13^C-NMR, FTIR and single-crystal X-ray diffraction. When available, characterization given in the literature was used for comparison. Chemical analysis of carbon, hydrogen, nitrogen and sulphur were performed by microanalytical methods using a Leco CHNS-932 analyzer. IR spectra were recorded on a Perkin-Elmer Spectrum 100 FTIR spectrophotometer, from KBr pellets in the 4000–400 cm^−1^ range, and on a Perkin-Elmer FTIR 1700X spectrophotometer, from Nujol mulls in the 500–150 cm^−1^ range. ^1^H, ^13^C NMR spectra were obtained with a Bruker Advance III 500 instrument at 500 MHz in DMF-d_7_. ^1^H NMR signals were referenced to residual proton resonances in deuterated solvents.

### 3.2. Synthesis of [PtCl_2_(PyTz)]·C_2_H_6_O (PtPyTz)

A solution of PyTz (96.5 mg, 0.5 mmol) in ethanol (30 mL) was added dropwise to a solution of [PtCl_2_(DMSO)_2_] (211 mg, 0.5 mmol) in ethanol (40 mL) at 50 °C and the mixture was refluxed overnight. Then, the resulting yellow solid was filtered off and washed with water and cold ether. From the resulting solution after filtration, yellow crystals were isolated after slow evaporation of ethanol. (192 mg, 76%). Anal. Calc. (%) for C_9_H_11_Cl_2_N_3_PtS·C_2_H_6_O: C, 26.13; H, 3.36; N, 8.31; S, 6.34 %. Found: C, 26.04; H, 3.25; N, 8.41; S, 6.53 %. IR (KBr): ν(NH) 3262, 3225, 3173, 3126, ν(C=N) 1631, (thiazine ring vibrations) 954, 794, 769, 735, 631, 594, (pyridine ring vibrations) 1599, 1527, 1479, 1421, 1349, 1318, 1268, 1100 (metal–ligand vibrations) 259, 252, 243, 232 cm^−1^. ^1^H NMR (500 MHz, DMF-*d*_7_) δ 11.20 (s, 1H), 9.01 (d, *J* = 6.3, Hz, 1H), 8.06 (t, *J =* 7.6 Hz, 1H), 7.39 (d, *J* = 8.2 Hz, 1H), 7.23 (t, *J =* 6.7 Hz, 1H), 4.02 (t, *J* = 5.5 Hz, 2H), 3.30 (t, *J* = 6.9 Hz, 2H), 2.09 (m, 2H). ^13^C NMR (126 MHz, DMF) δ 156.0, 150.6, 148.7, 140.6, 120.5, 114.2, 52.4, 27.2, 23.4.

### 3.3. Synthesis of [PdCl_2_(PyTz)]·C_2_H_6_O (PdPyTz)

A solution of PyTz (96.5 mg, 0.5 mmol) in ethanol (30 mL) was added dropwise to a solution of Na_2_[PdCl_4_]·H_2_O (156 mg, 0.5 mmol) in ethanol (40 mL), and the mixture was refluxed overnight. Then, the resulting orange solid was filtered off and washed with water and cold ether. From the resulting solution after filtration, orange crystals were isolated after slow evaporation of ethanol. (182.2 mg, 87%). Anal. Calc. (%) for C_9_H_11_Cl_2_N_3_PdS·0.75C_2_H_6_O·0.75H_2_O: C, 30.10; H, 4.06; N, 10.03; S, 7.66 %. Found: C, 29.82; H, 3.72; N, 9.81; S, 7.44 %. IR (KBr): ν(NH) 3261, 3226, 3175, 3124, ν(C=N) 1629, (thiazine ring vibrations) 952, 795, 767, 734, 630, 595, (pyridine ring vibrations) 1599, 1526, 1475, 1420, 1351, 1315, 1095 (metal–ligand vibrations) 254, 242, 233 cm^−1^. ^1^H NMR (500 MHz, DMF-*d*_7_) δ 11.33 (s, 1H), 8.75 (d, *J* = 5.9 Hz, 1H), 8.06 (t, *J* = 7.6 Hz, 1H), 7.41 (d, *J* = 8.3 Hz, 1H), 7.29 (t, *J* = 6.6 Hz, 1H), 3.85 (t, *J* = 5.4 Hz, 2H), 3.29 (t, *J* = 6.8 Hz, 2H), 2.05 (m, 2H). ^13^C NMR (126 MHz, DMF) δ 162.1, 156.78, 151.1, 148.2, 141.5, 120.3, 114.3, 51.9, 27.2, 23.2.

### 3.4. Isolation of Single Crystals of PyTzHCl·2H_2_O

2-(2-pyridyl)iminotetrahydro-1,3-thiazinehydrochloride–water (1/2) (PyTzHCl·2H_2_O) was synthetized according to a reported method [24]. However, the crystalline structure of this compound has not been previously reported in the literature. Therefore, in order to obtain single crystals of PyTzHCl·2H_2_O, the solid obtained from the synthesis was recrystallized by slow diffusion of n-hexane in a solution of PyTzHCl·2H_2_O in ethanol 96%, which afforded colourless single crystals of PyTHCl·2H_2_O after 10 days. Anal. Calc. (%) for C_9_H_16_ClN_3_O_2_S: C, 40.83; H, 6.29; N, 15.81; S, 11.56 %. Found: C, 40.67; H, 6.02; N, 15.80; S, 12.07 %.

### 3.5. X-ray Diffraction

Crystals of the precursor PyTzHCl·2H_2_O and the complexes PtPyTz and PdPyTz were obtained as mentioned above. Crystal, structure determination and refinement data are reported in Table 4.

Data were collected on a Bruker Kappa APEXII CCD diffractometer, using graphite-monochromated Mo-Kα radiation (λ = 0.71073 Å). All data were corrected for Lorentz and polarization effects, while absorption corrections were performed by means of SADABS [42] program. The structures were solved by direct methods and subsequent Fourier differences using the SHELXS-14 [43] program and refined by full-matrix least-squares on F2 with SHELXL-18 [44], included in WINGX [45] package, assuming anisotropic displacement parameters for non-hydrogen atoms. All hydrogen atoms attached to carbon or nitrogen atoms were positioned geometrically, with U_iso_ values derived from U_eq_ values of the corresponding carbon and nitrogen atoms. Hydrogen atoms of the water molecules were detected by Fourier differences and were refined with isotropic temperature factors. Crystallographic data for precursor PyTzHCl·2H_2_O and for complexes PtPyTz and PdPyTz were deposited at the CCDC (Cambridge Crystallographic Data Center, Cambridge, UK) with CCDC numbers 2055561, 2055559 and 2055560, respectively.

### 3.6. Cell Culture and Treatments

Cells were grown in DMEM (HeLa), McCoy’s 5a (SK-OV-3), RPMI-1640 (U-937 and HL-60) or DMEM F-12 (MCF10A) medium. The media were supplemented with 2 mM L-glutamine, 10% heat-inactivated FBS, 100 U/mL penicillin, and 10 μg/mL streptomycin, except for DMEM F-12, which was supplemented with 5% horse serum, 0.5 µg/mL hydrocortisone, 10 µg/mL insulin, 20 ng/mL EGF, 100 ng/mL cholera toxin, 100 U/mL penicillin, and 10 μg/mL streptomycin. Cells were cultured under a humidified atmosphere containing 95% air and 5% CO2 at 37 °C. Cells were usually plated at a density of 7 × 10^5^ cells/mL (HeLa, SK-OV-3 and MCF10A) or 3 × 10^5^ cells/mL (HL-60 and U-937) into 25 cm^2^ culture flasks and the viability was routinely kept at >95% as assayed by the Trypan-blue exclusion method. Cells were treated with the free ligand (PyTz), its Pt(II) and Pd(II) complexes (PtPyTz and PdPyTz) or vehicle for 24 h at the indicated concentrations. DMF was used as vehicle and its final concentration did not exceed 0.5% (*v*/*v*).

### 3.7. In Vitro Cytotoxicity Assay

The cytotoxic effects of the different compounds were evaluated on all four cell lines by means of the CellTiter 96 AQueous One Solution Cell Proliferation Assay, which is based on the reduction of an MTS tetrazolium compound. Cells were seeded in 96-well plates at a density of 8 × 10^3^ cells/well (HeLa and MCF10A), 6 × 10^3^ cells/well (SK-OV-3), 1.5 × 10^4^ cells/well (U-937) or 2.5 × 10^4^ cells/well (HL-60). After treating cultures for 24 h, assays were performed by adding 10 µL of the CellTiter 96 AQueous One Solution Reagent directly to culture wells, incubating for 15 min (HeLa), 30 min (SK-OV-3), 1 h (U-937 and MCF10A) or 2 h (HL-60) at 37 °C, and then recording absorbance on a microplate reader (Infinite M200; Tecan Gorup Ltd., Männedorf, Switzerland) at both a test wavelength of 490 nm and a reference wavelength of 650 nm to subtract background. All analyses were run in triplicate. The cell viability was calculated as percentage of control values (untreated samples).

### 3.8. Statistical Analysis

Data are presented as mean ± standard deviation (SD). To compare the different treatments, statistical significance was calculated by one-way analysis of variance (ANOVA) followed by Tukey’s test. IC_50_ values obtained from the dose–response curve of each compound were calculated by fitting the curve to the data using nonlinear regression to generate a four-parameter sigmoid dose–response equation. *p* < 0.05 was considered to indicate a statistically significant difference. The statistics software used was GraphPad Prism 7.04 for Windows.

## 4. Conclusions

New platinum(II) and palladium(II) complexes containing a thiazine-pyridine derivative ligand, PyTz, were prepared and characterized. It should be noted that PdPyTz is the first crystal structure that is described with Pd(II) linked to a nitrogen atom belonging to a 1,3-thiazine heterocycle. With regard to the antiproliferative activity, our findings show that the newly developed chemotherapeutic agent PtPyTz has greater cytotoxic activity than the PyTz-containing Pd(II) complex and the PyTz free ligand in all four human tumor cell lines tested, being more effective in leukemic cell lines than in those derived from solid tumors and non-tumor cell line. This suggests that the cytotoxic effect of these complexes is modulated by the central metal.

## Figures and Tables

**Table 1:** Selected bond lengths (Å), angles (°) and hydrogen-bond parameters for PyTzHCl·2H_2_O.

S(1)-C(1)	1.744(2)	S(1A)-C(1A)	1.873(6)
C(1)-N(1)	1.302(2)	C(1A)-N(1A)	1.229(8)
C(1)-N(2)	1.355(2)	C(1A)-N(2A)	1.408(5)
N(1)-C(2)	1.396(7)	N(1A)-C(2A)	1.558(7)
N(2)-C(5)	1.401(2)	N(2A)-C(5A)	1.415(9)
N(3)-C(5)	1.328(2)	N(3A)-C(5A)	1.312(12)
N(3)-C(9)	1.322(2)	C(9)-C(8)	1.382(2)
S(1)-C(1)-N(1)	125.5(1)	S(1A)-C(1A)-N(1A)	128.4(3)
N(1)-C(1)-N(2)	122.0(1)	N(1A)-C(1A)-N(2A)	124.7(4)
N(2)-C(5)-N(3)	118.4(1)	N(2A)-C(5A)-N(3A)	121.1(7)
N(3)-C(5)-C(6)	122.9(1)	C(5A)-N(3A)-C(6)	119.5 (7)
**D-H···A**	**A position**	**D···A (Å)**	**D-H···A (°)**
N(2)-H(2)···O(2W)	x, y, z	2.701(2)	168.7(1)
N(1)-H(1)··· N(3)	x, y, z	2.647(2)	133.7(1)
O(2W)-H(4W)···O(1W)	x, y, z	2.761(2)	173.6(2)
O(2W)-H(3W)···Cl(1)	−x, −y + 1, −z + 1	3.177(1)	173.9(2)
O(1W)-H(2W)···Cl(1)	x, −y−1, +z−1	3.162(1)	173.5(1)
O(1W)-H(1W)···Cl(1)	−x+1, −y + 1, −z + 1	3.209(1)	171.8(2)

**Table 2:** Selected bond lengths (Å), angles (°) and hydrogen-bond parameters for PtPyTz and PdPyTz.

PtPyTz	Pt-Cl(1)	2.306(1)	Pt-Cl(2)	2.306
	Pt-N(1)	2.013(2)	Pt-N(3)	2.017(2)
	Cl(1)-Pt-Cl(2)	90.1(1)	Cl(1)-Pt-N(1)	177.9(1)
	Cl(1)-Pt-N(3)	91.5(1)	Cl(2)-Pt-N(1)	91.0(1)
	Cl(2)-Pt-N(3)	177.5(1)	N(1)-Pt-N(3)	87.4(1)
	**D-H···A**	**A position**	**D···A (Å)**	**D-H···A(°)**
	N(2)-H(2)···O	x, y, z	2.736(3)	162.5(2)
	O-H(0)··· Cl(2)	x, y +1, z+1/2	3.136(2)	135.3(2)
PdPyTz	Pd-Cl(1)	2.302(1)	Pd-Cl(2)	2.303(1)
	Pd-N(1)	2.016(1)	Pd-N(3)	2.021(1)
	Cl(1)-Pd-Cl(2)	90.6(2)	Cl(1)-Pd-N(1)	177.7(2)
	Cl(1)-Pd-N(3)	91.5(1)	Cl(2)-Pd-N(1)	90.8(1)
	Cl(2)-Pd-N(3)	176.2(1)	N(1)-Pd-N(3)	87.1(1)
	**D-H···A**	**A position**	**D···A (Å)**	**D-H···A(°)**
	N(2)-H(2)···O	x, y, z	2.744(2)	175.8(2)
	O-H(0)···Cl(2)	x, y−1, z	3.180(3)	162.1(1)

**Table 3:** Cytotoxicity (IC50 ± SD, µM) of the free ligand PyTz and their Pt(II) and Pd(II) complexes towards selected tumor and healthy (non-tumor) cell lines.

	U-937	HL-60	HeLa	SK-OV-3	MCF10A
**PtPyTz**	26.36 ± 2.56 ^b^	39.25 ± 3.86 ^b^	63.38 ± 5.63 ^b^	77.97 ± 6.36 ^b^	75.94 ± 7.74 ^b^
**PdPyTz**	90.96 ± 17.66 ^c^	115.20 ± 17.01 ^c^	78.62 ± 8.21 ^c^	117.20 ± 11.69 ^c^	81.54 ± 8.29 ^c^
**PyTz**	79.42 ± 14.64 ^c^	107.10 ± 15.78 ^c^	82.31 ± 9.41 ^c^	97.32 ± 10.05 ^c^	101.70 ± 2.00 ^d^
**Cisplatin**	7.89 ± 0.54 ^a^	11.32 ± 1.04 ^a^	16.08 ± 1.01 ^a^	55.41 ± 3.04 ^a^	50.34 ± 5.16 ^a^

Within each column, values followed by the same lowercase letter are not significantly different (*p* < 0.05; Tukey’s test).

**Table 4:** Crystal data, data collection and refinement details for PyTzHCl·2H_2_O, PtPyTz and PdPyTz.

	PyTzHCl·2H_2_O	PtPyTz	PdPyTz
Crystal shape	Plate	Plate	Prism
Colour	Colourless	Yellow	Orange
Size (mm)	0.59 × 0.30 × 0.11	0.17 × 0.13 × 0.07	0.36 × 0.33 × 0.14
Chemical formula	C_9_H_16_N_3_OS	C_11_H_17_Cl_2_N_3_OPtS	C_11_H_17_Cl_2_N_3_OPdS
Formula weight	265.76	505.32	416.63
Crystal system	Triclinic	Monoclinic	Monoclinic
Space group	P-1	P2_1_/c	P2_1_/c
Unit cell dimensions			
a (Å)	6.9676(2)	12.6205(4)	12.6048(4)
b (Å)	9.5773(3)	9.0616(2)	9.0445(3)
c (Å)	9.5927(3)	13.8145(4)	13.8167(5)
α (°)	96.001(2)		
β (°)	97.830(2)	109.540(2)	109.575(2)
γ (°)	101.185(2)		
Cell volume (Å^3^)	616.44(4)	1488.8(7)	1484.12(9)
Z	2	4	4
Dcalc (g cm^−3^)	1.432	2.254	1.865
μ (mm^−1^)	0.47	9.918	1.746
F(000)	280	960	832
θ range	2.16–35.57	2.74–32.26	2.74–30.29
Index ranges	−11≤ h ≤ 11,	−19 ≤ h ≤ 19,	−19 ≤ h ≤ 19,
	−16 ≤ k ≤ 16,	−13 ≤ k ≤ 13,	−13 ≤ k ≤ 13,
	−16 ≤ l ≤ 16	−21 ≤ l ≤ 21	−21 ≤ l ≤ 21
Temperature (K)	100	100	100
Independent reflections	6033	5660	5649
Observed reflections	5254 [F > 4.0 σ(F)]	4407 [F > 4.0 σ(F)]	4746 [F > 4.0 σ(F)]
No. of refined parameters	195	180	181
R [F > 4.0 σ(F)]	0.042	0.030	0.027
wR [F > 4.0 σ(F)]	0.125	0.044	0.051
GOF	1.052	0.993	1.021
ρmax, ρmin (e Å^−3^)	0.622, −1,210	1.604, −1.464	0.545, 1.034

## Data Availability

Data is contained within the article or supplementary material. Anyway, the datasets generated during the current study are available from the corresponding author on request.

## Supplementary Materials

The following materials are available online at https://www.mdpi.com/article/10.3390/ph14050395/s1. Figure S1: Structural parameters of {[(H_2_O)_5_Cl_3_]^3−^}_n_ tapes in PyTzHCl·2H_2_O title; Figure S2: Supramolecular arrangement in PtPyTz stabilized by intramolecular hydrogen-bonds; Figure S3: Supramolecular arrangement in PdPyTz stabilized by intramolecular hydrogen-bonds; Table S1: ^1^H NMR spectral data for PyTz and its Pt (II) and Pd(II) complexes in DMF-d_7_ solvent; Figure S4: ^1^H NMR spectrum of PyTz in DMF-d_7_; Figure S5: ^13^C NMR spectrum of PyTz in DMF-d_7_; Figure S6: ^1^H NMR spectrum of PtPyTz in DMF-d_7_; Figure S7: ^13^C NMR spectrum of PtPyTz in DMF-d_7_; Figure S8: ^1^H NMR spectrum of PdPyTz in DMF-d_7_; Figure S9: ^13^C NMR spectrum of PdPyTz in DMF-d_7_; Figure S10. ^1^H NMR spectrum of PyTz in D_2_O:DMF-d_7_ (11:1 ratio) after preparation (red), 1 h (green), 6 h (blue), 24 h (purple); Figure S11. ^1^H NMR spectrum of PtPyTz in D_2_O:DMF-d_7_ (11:1 ratio) after preparation (red), 1 h (green), 6 h (blue), 24 h (purple); Figure S12. ^1^H NMR spectrum of PyTz in D_2_O:DMF-d_7_ (11:1 ratio) after preparation (red), 1 h (green), 6 h (blue), 24 h (purple); Figure S13: FTIR spectrum of PtPyTz between 4000–400 cm^−1^; Figure S14: FTIR spectrum of PtPyTz between 500–150 cm^−1^; Figure S15: FTIR spectrum of PdPyTz between 4000–400 cm^−1^; Figure S16: FTIR spectrum of PdPyTz between 500–150 cm^−1^; Table S2: ADME properties factor of the synthesized compounds.
