# Supplementary material for: Synthesis, Characterization and Antiproliferative Evaluation of Pt(II) and Pd(II) Complexes with a Thiazine-Pyridine Derivative Ligand
Source: Pharmaceuticals (Basel). 2021 Apr 22;14(5):395. doi: 10.3390/ph14050395 (PMC8143583; doi:10.3390/ph14050395)
Supplement: Supplementary file 1 [file pharmaceuticals-14-00395-s001.zip › pharmaceuticals-1142730-supplementary.pdf]

Supporting Information

Synthesis, characterization and antiproliferative evaluation of Pt(II) and Pd(II) complexes with a thiazine-pyridine derivative ligand

Silvia Gutiérrez-Tarriño[1,#], Javier Espino[2,#,*], Francisco Luna-Giles[1], José A. Pariente[2], Ana B. Rodríguez[2], Emilio Viñuelas-Zahínos[1,*]

[1] Department of Organic and Inorganic Chemistry (Chemistry of Coordination Research Group), Faculty of Science, University of Extremadura, Badajoz, Spain;

[2] Department of Physiology (Neuroimmunophysiology and Chrononutrition Research Group), Faculty of Science, University of Extremadura, Badajoz, Spain;

# Both authors contributed equally.

** Correspondence: emilvin@unex.es, (E.V.-Z.); jespino@unex.es, (J.E.)*

This manuscript is in Memoriam of Prof. Álvaro Bernalte García, Group Leader of the Coordination Chemistry Research Group

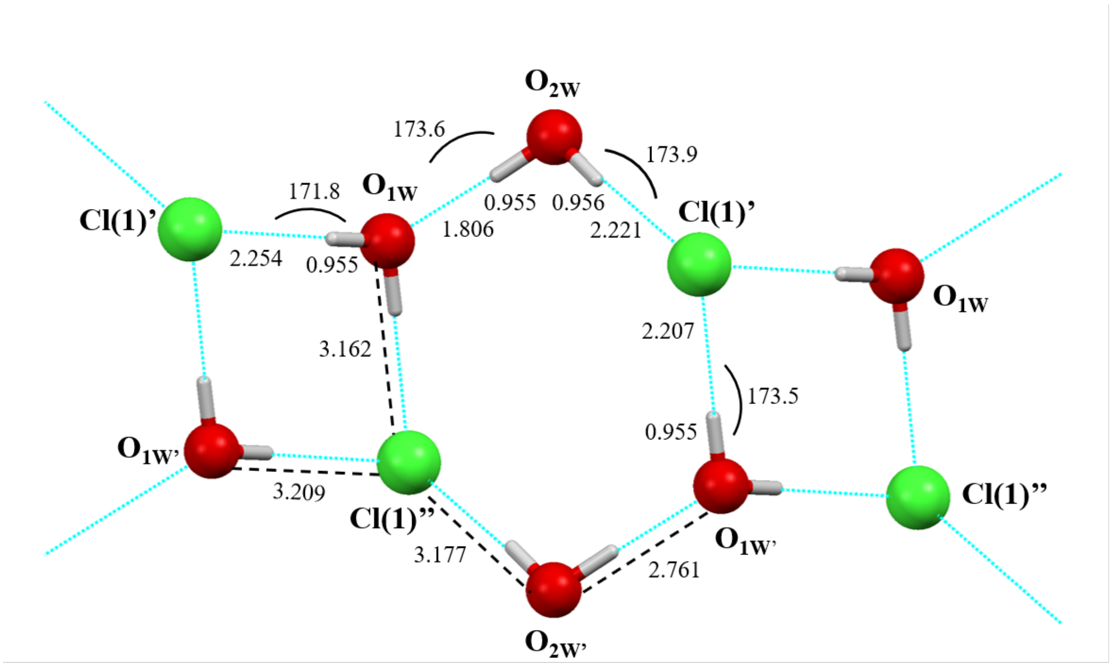

Figure S1. Structural parameters of $\{[(H_2O)_5Cl_3]^{3-}\}_n$ tapes in $PyTzHCl{\cdot}2H_2O$.

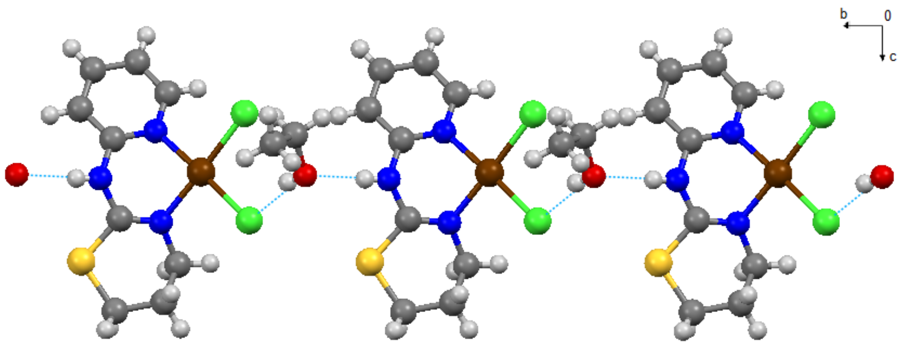

Figure S2. Supramolecular arrangement in PtPyTz stabilized by intramolecular hydrogen-bonds.

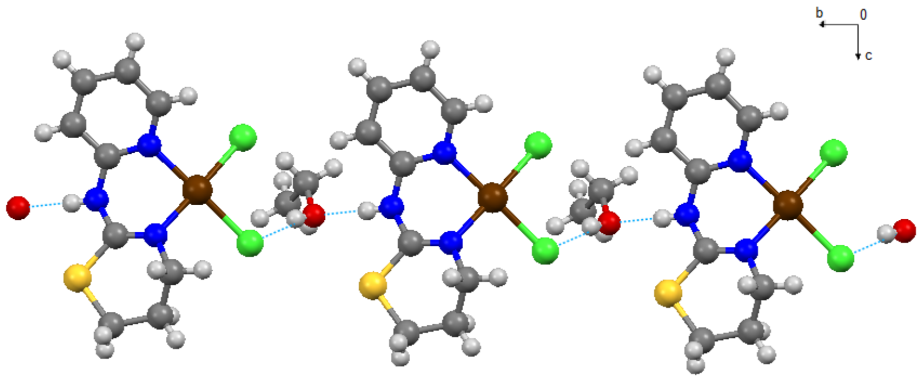

Figure S3. Supramolecular arrangement in PdPyTz stabilized by intramolecular hydrogen-bonds.

Table S1. ^{1}H NMR spectral data for PyTz and its Pt(II) and Pd(II) complexes in DMF-d_7 solvent.

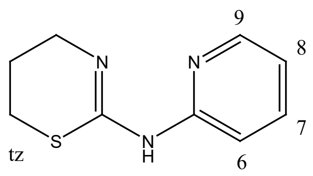

Compound	N-H	H(9)	H(7)	H(8)	H(6)	N-CH$_2$ (tz)	S-CH$_2$(tz)	CH$_2$ (tz)
**PyTz**	11.31	8.17	7.63	6.95	6.86	3.56	3.12	2.05
**PtPyTz**	11.20	9.01	8.06	7.23	7.39	4.02	3.30	2.09
**PdPyTz**	11.33	8.75	8.06	7.29	7.41	3.85	3.29	2.05

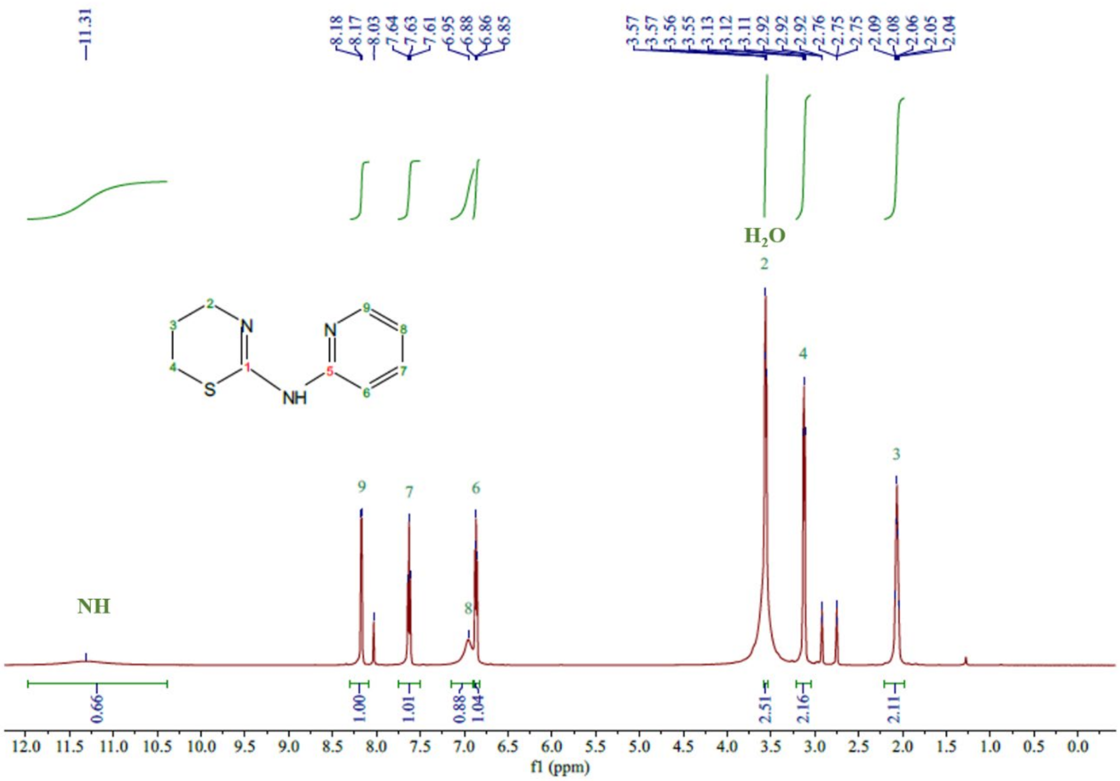

Figure S4. ^{1}H NMR spectrum of PyTz in DMF-d_7.

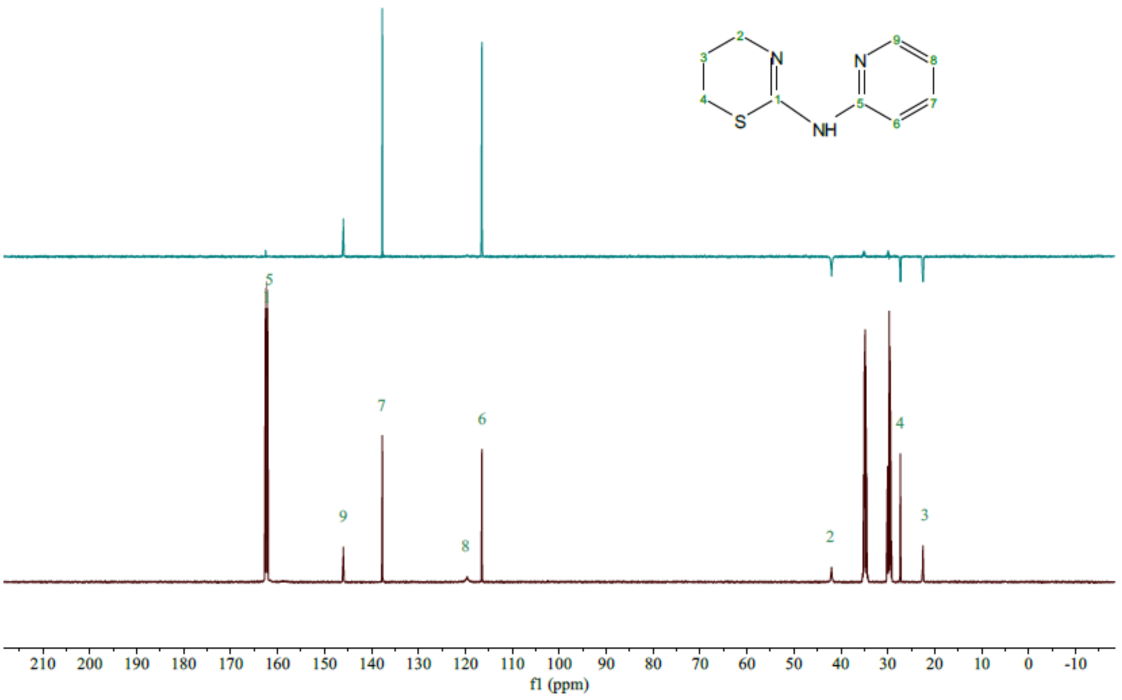

Figure S5. ^{13}C NMR spectrum of PyTz in DMF-d_7.

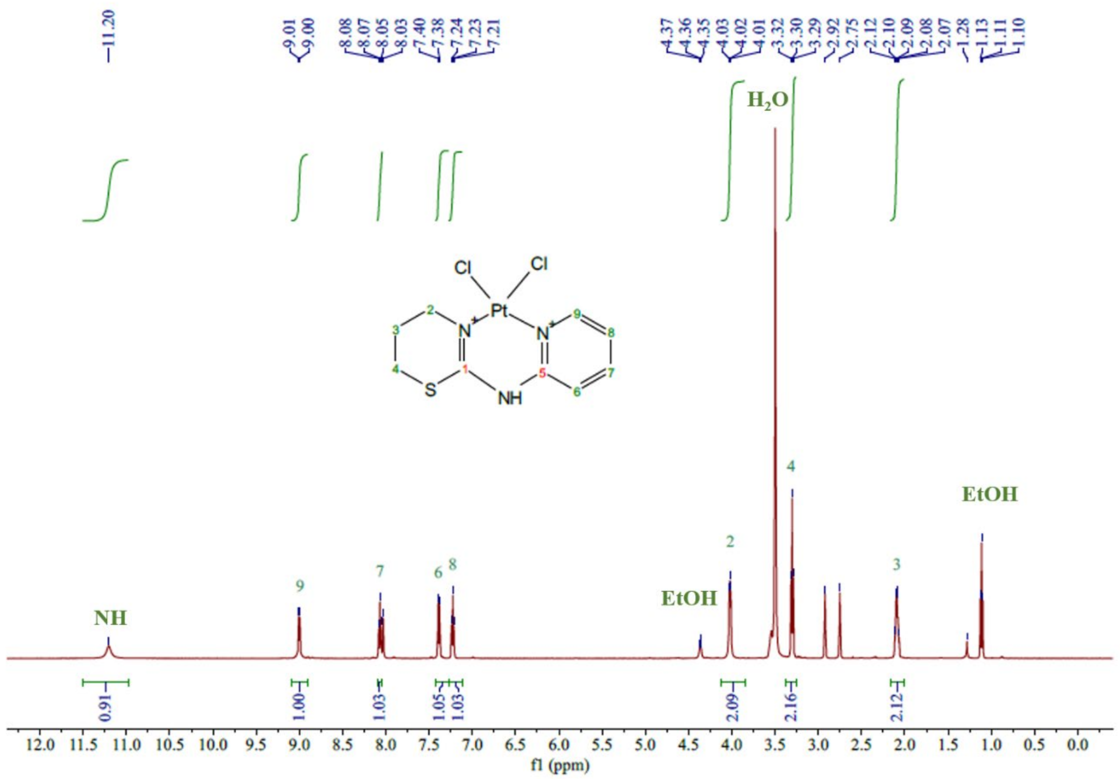

Figure S6. ^{1}H NMR spectrum of PtPyTz in DMF-d_7.

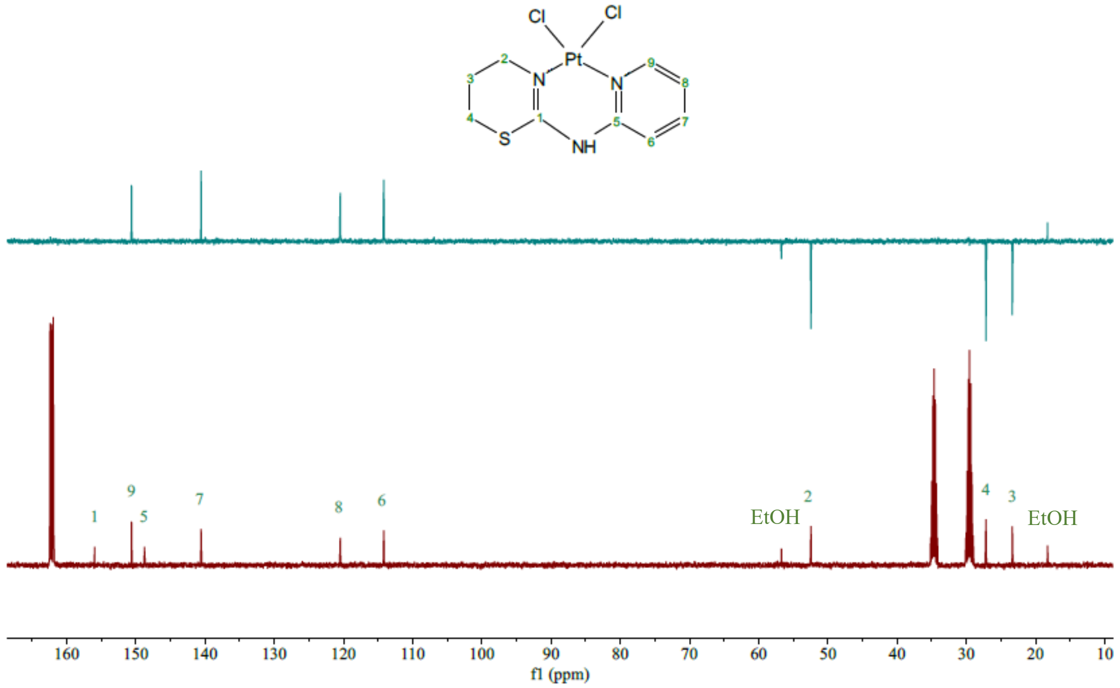

Figure S7. ^{13}C NMR spectrum of PtPyTz in DMF-d_7.

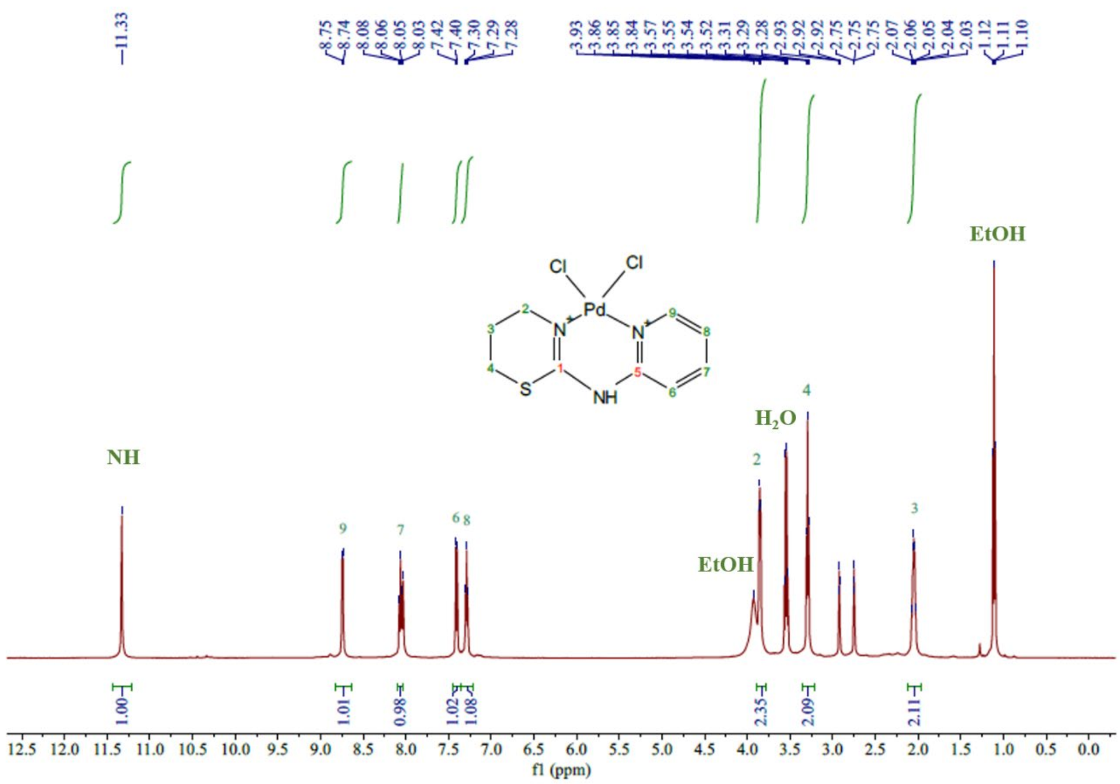

Figure S8. ^{1}H NMR spectrum of PdPyTz in DMF-d$_7$.

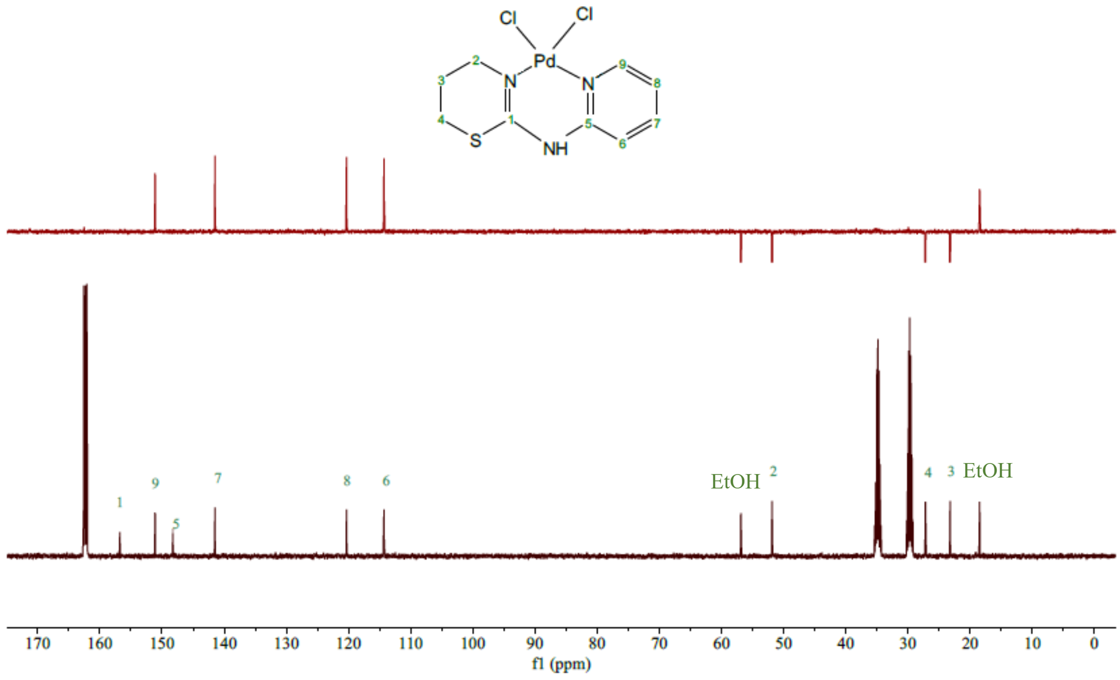

Figure S9. ^{13}C NMR spectrum of PdPyTz in DMF-d$_7$.

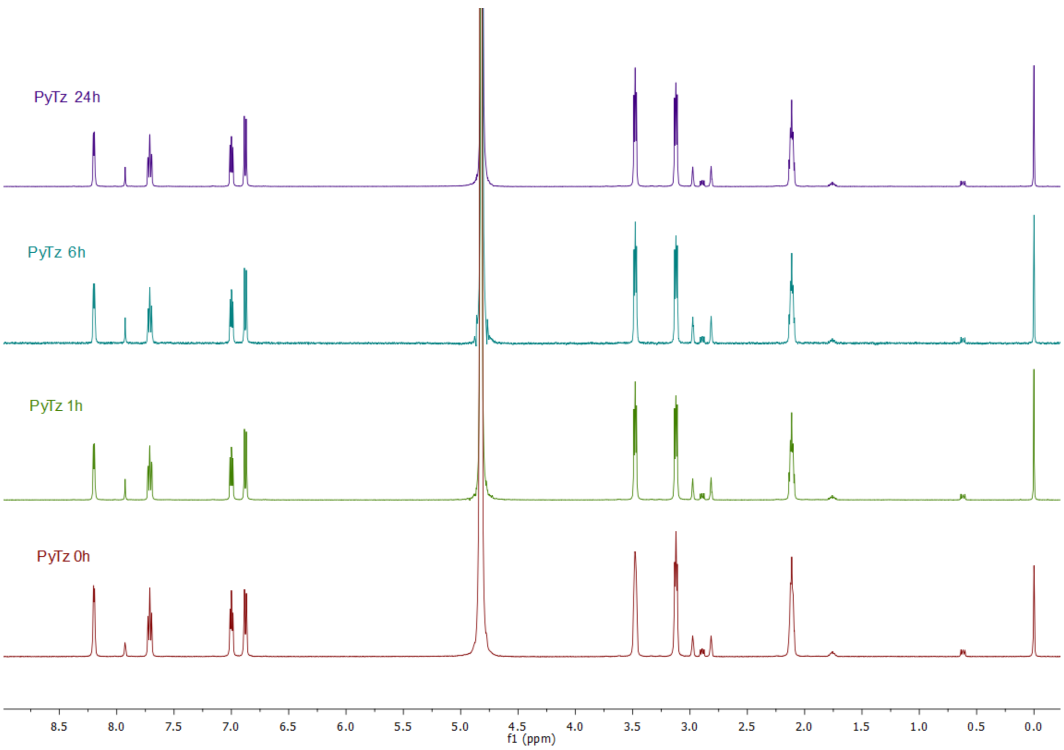

Figure S10. ^{1}H NMR spectrum of PyTz in D_2O:DMF-d_7 (11:1 ratio) after preparation (red), 1 hour (green), 6 hours (blue), 24 hours (purple).

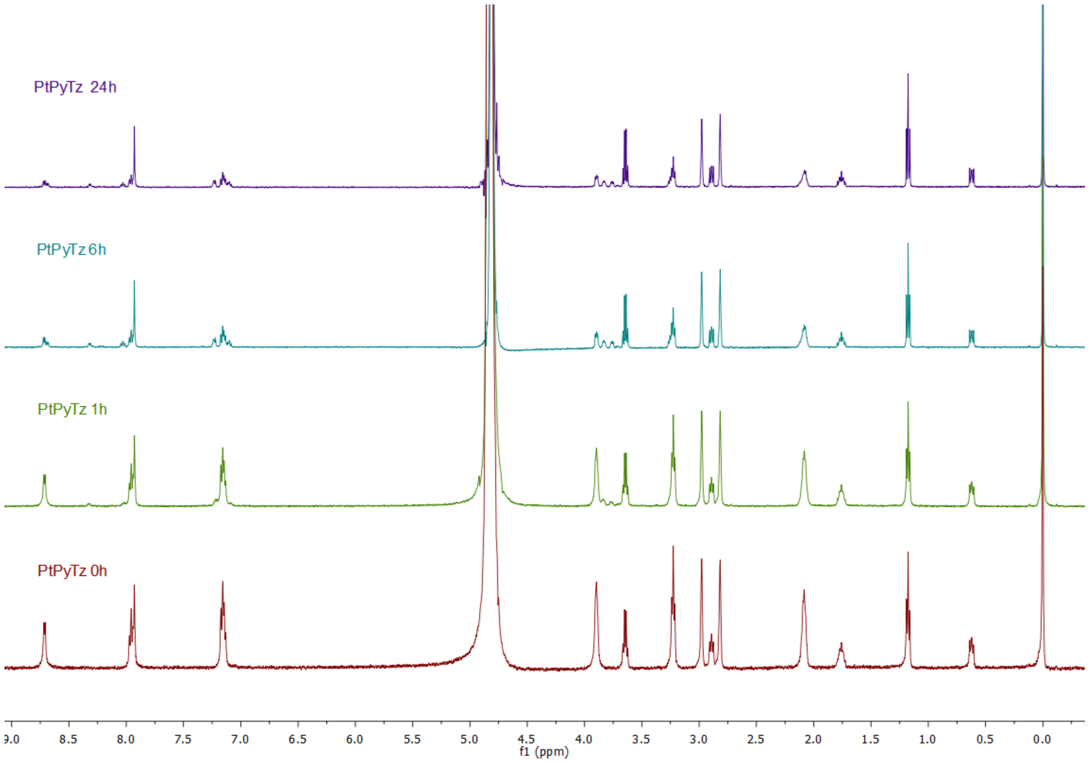

Figure S11. ^{1}H NMR spectrum of PtPyTz in D_2O:DMF-d_7 (11:1 ratio) after preparation (red), 1 hour (green), 6 hours (blue), 24 hours (purple).

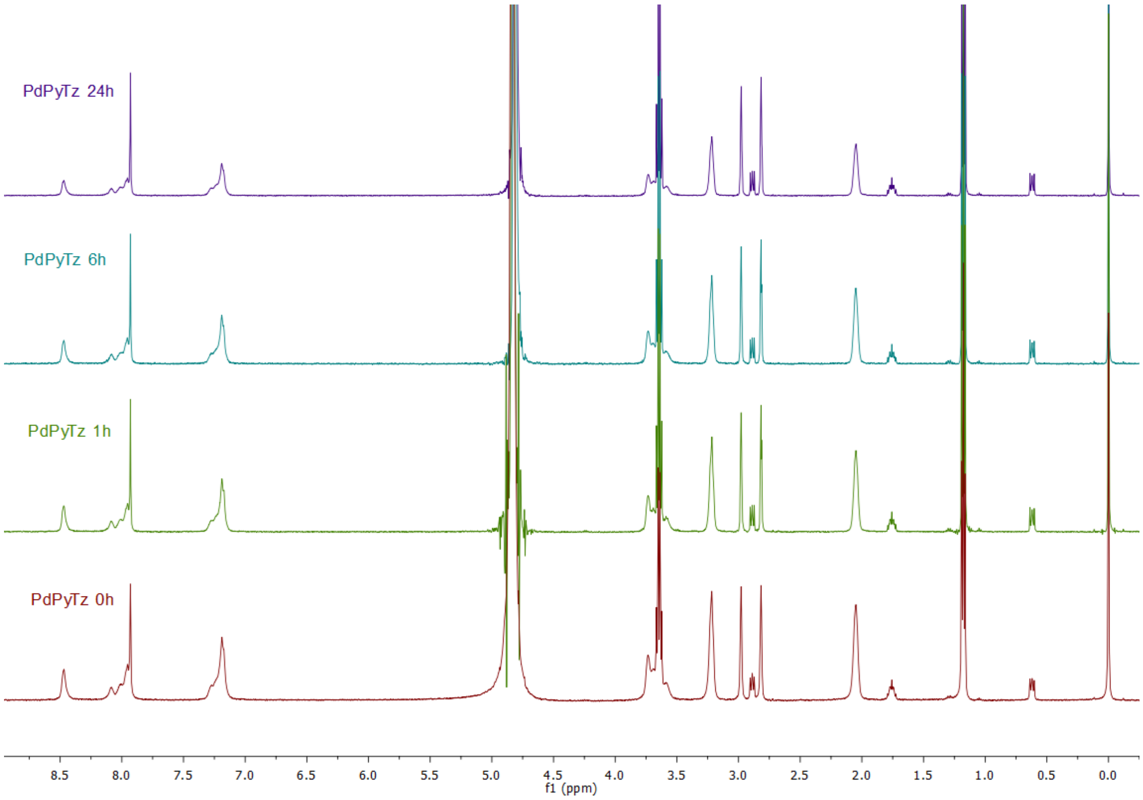

Figure S12. ^{1}H NMR spectrum of PdPyTz in D_2O:DMF-d_7 (11:1 ratio) after preparation (red), 1 hour (green), 6 hours (blue), 24 hours (purple).

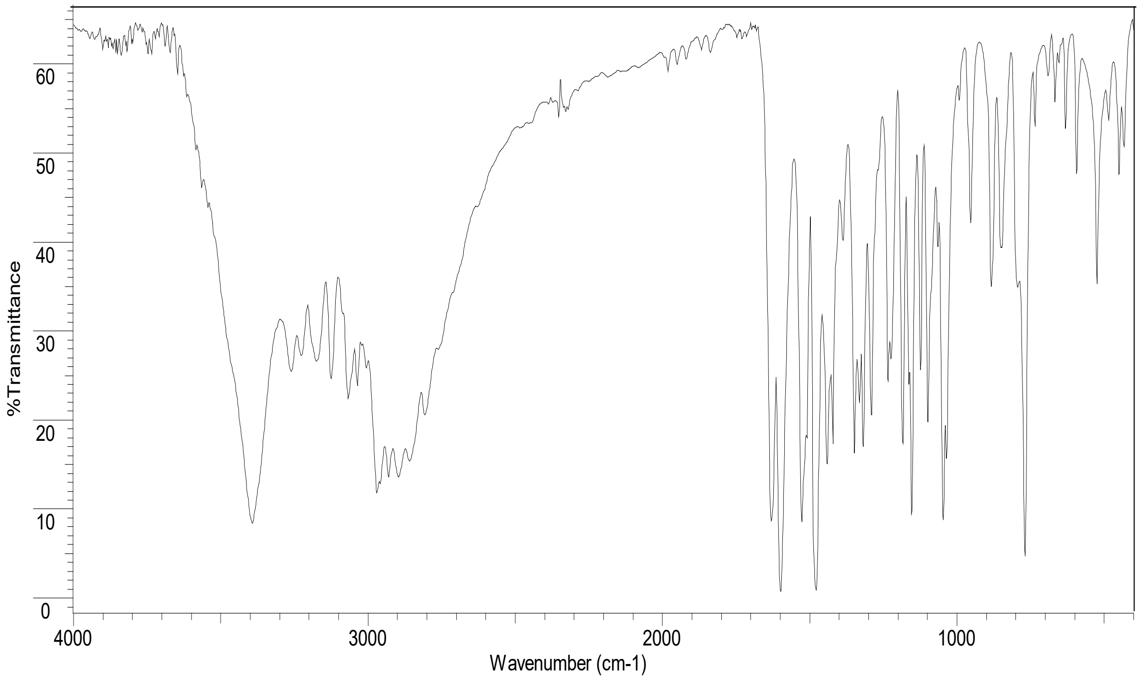

Figure S13. FTIR spectrum of PtPyTz between 4000-400 cm^{-1}.

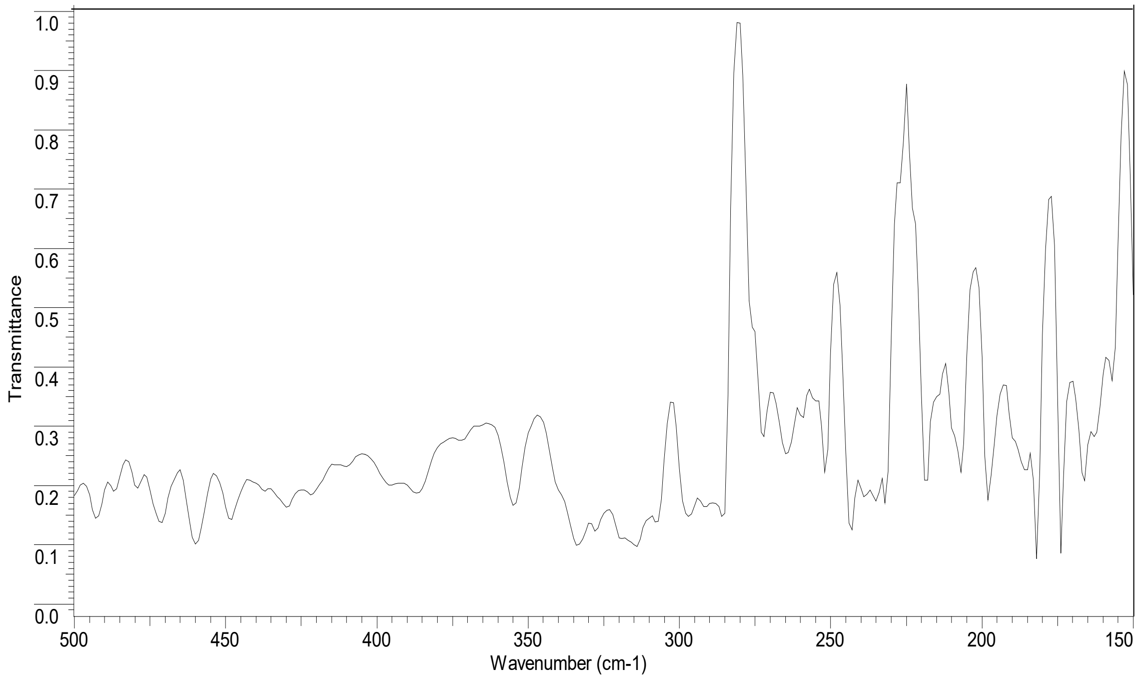

Figure S14. FTIR spectrum of PtPyTz between 500-150 cm^{-1}.

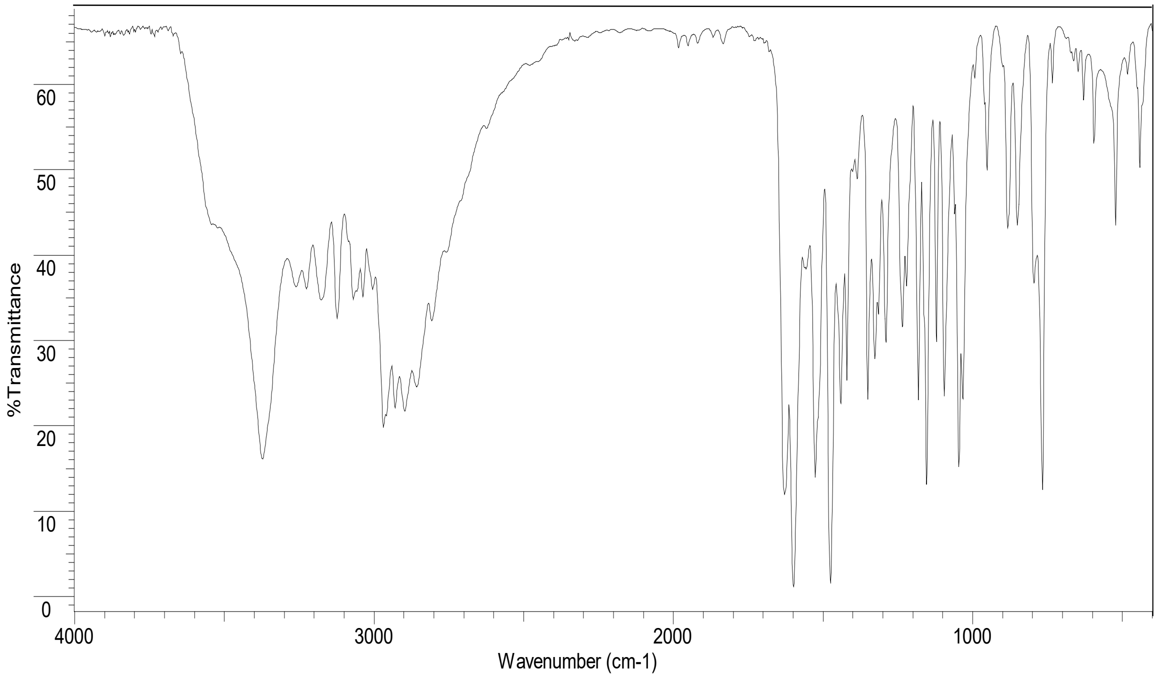

Figure S15. FTIR spectrum of PdPyTz between 4000-400 cm^{-1}.

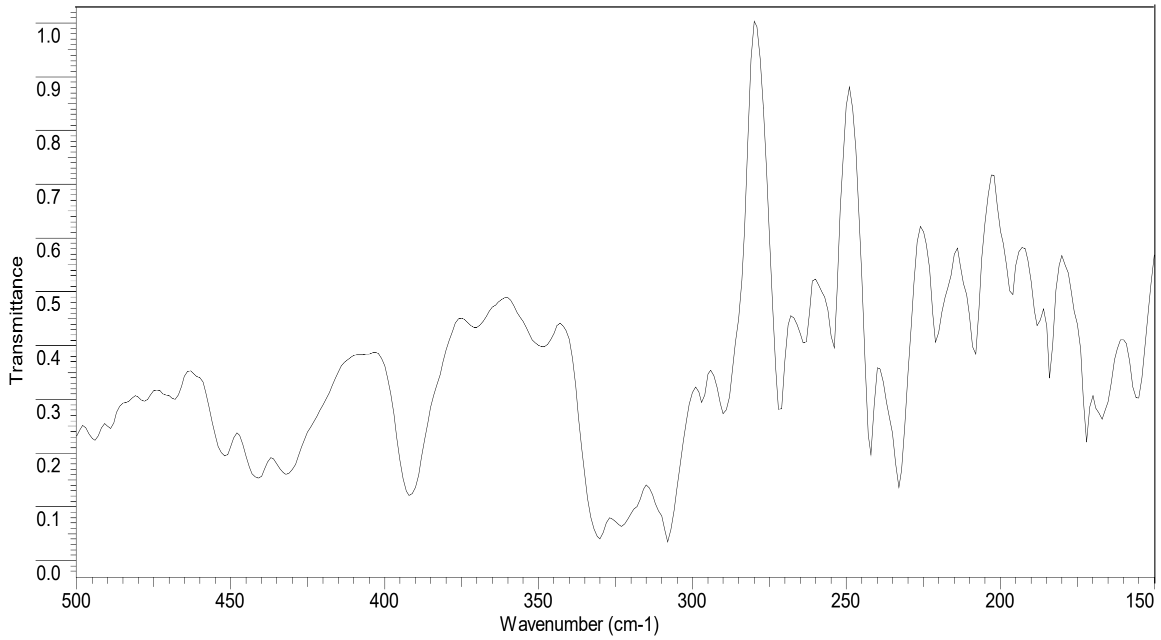

Figure S16. FTIR spectrum of PdPyTz between 500-150 cm^{-1}.

Table S2. ADME properties factor of the synthesized compounds.

	PyTz	PtPyTz	PdPyTz
**Mw(g/mol)**	193.27	459.25	370.59
**TPSA**[1]	62.58	54.62	54.62
**LogP$_{o/w}$**[2]	1.54	1.59	1.48
**LogS**[3]	-2.10	-4.69	-4.41
**Rotable bonds**	2	0	0
**GI Absorption**[4]	High	High	High
**BBB permeant**[4]	Yes	Yes	Yes
**Lipinski violiations**[5]	0	0	0
**PAINS alerts**[6]	0	0	0

1. Topological Polar Surface Area calculated from: Etrl, P.; Rohde, B.; Selzer, P. Fast calculation of molecular polar surface area as a sum of fragment-based contributions and its application to the prediction of drug transport properties. J. Med. Chem. **2000**, *43*, 3714–3717. 2.

2. Consensus Log $P_{o/w}$ average of 5 prediction methods.

3. ESOL topological method implemented from Delaney, J. S. Prediction of aqueous solubility and partition coefficient optimized by a genetic algorithm based descriptor selection method. J. Chem. Inf. Model. **2004**, *44*, 1000-1005.

4. Saina, A.; Zoete, V. A BOILED-Egg to predict gastrointestinal absorption and brain penetration of small molecules. Chem. Med. Chem. **2016**, *11*, 1117-1121.

5. Lipinski, C. A.; Lombardo, F.; Dominy, B. W.; Feeney, P. J. Experimental and computational approaches to estimate solubility and permeability in drug discovery and development settings. Adv. Drug Deliv. Rev. **2001**, *46*, 3-26.

6. Baell, J.B.; Holloway G. A. New substructure filters for removal of pan assay interference compounds (PAINS) from screening libraries and for their exclusion in bioassays. J. Med. Chem. **2010**, *53*, 2719-2740.
